# Prediction of Ultra-High-Performance Concrete (UHPC) Compressive Strength Based on Convolutional Neural Networks

**DOI:** 10.3390/ma18122851

**Published:** 2025-06-17

**Authors:** Li Xu, Xiaochun Yu, Chenhui Zhu, Ling Wang, Jie Yang

**Affiliations:** 1 Jiangsu Vocational College of Business, Nantong 226000, China; 2014231@jsbc.edu.cn; 2 School of Transportation and Civil Engineering, Nantong University, Nantong 226000, China; 2333310001@stmail.ntu.edu.cn (X.Y.); zch198916@ntu.edu.cn (C.Z.); 3 Nantong Vocational University, Nantong 226000, China

**Keywords:** UHPC, convolutional neural network, compressive strength, prediction model

## Abstract

This paper investigates the potential of deep learning in predicting the compressive strength of ultra-high-performance concrete (UHPC) by developing a convolutional neural network (CNN) model with two convolutional layers. The proposed CNN architecture demonstrates capability in accurately predicting UHPC compressive strength from tabular data encompassing various material compositions. Ten input variables were selected, including the cement content, water content, silica fume content, silica powder content, silica sand content, superplasticizer content, and curing parameters. The model was trained and tested on a dataset comprising 219 samples. Experimental results indicated excellent predictive performance, with the CNN achieving a coefficient of determination (*R*^2^) of 0.959 on the test set and a mean absolute percentage error (MAPE) of 5.55%, demonstrating both accuracy and stability. Comparative analysis revealed that the CNN’s performance was comparable to established machine learning methods like XGBoost (*R*^2^ = 0.961), which are typically more suited for tabular data. Furthermore, SHAP (SHapley Additive exPlanations) analysis confirmed the model’s interpretability. These findings collectively suggest that the CNN-based approach shows considerable promise for predicting compressive strength across diverse UHPC formulations.

## 1. Introduction

UHPC, a novel composite material with superior performance, shows promising application potential [1,2]. Despite systematic analysis in existing research on its applications and various proposed improvements, UHPC faces numerous challenges in practical applications, including high costs. The preparation materials and processes involved still require further exploration [3,4,5]. Among many considerations, compressive strength is a crucial performance indicator of UHPC. Studying the variation in UHPC compressive strength with different materials and preparation methods, and predicting its strength based on these insights, is essential for improving UHPC design and construction quality.

Current research on the mechanical properties of UHPC commonly employs various materials including magnetite [6] and recycled aggregates [7,8] to achieve different performance and cost requirements [9,10,11]. These studies systematically investigate the underlying mechanisms through which these materials affect mechanical properties at both macroscopic and microscopic scales.

Macroscopic studies on UHPC’s mechanical properties have identified silicon-containing materials and fibers as critical influencing factors [12,13]. Research has demonstrated that fiber characteristics—including the type [14,15,16,17], volume fraction, morphology, and aspect ratio [18,19,20]—along with parameters of silicon-containing materials such as the dosage [21,22,23], incorporation methods [24], and curing regimes [25], significantly impact UHPC’s compressive strength and other mechanical performance indicators.

Microstructural analyses employing low-field nuclear magnetic resonance, backscattered electron imaging, X-ray diffraction, and scanning electron microscopy [26,27] have revealed the crucial role of material microstructure in governing how fibers and silicon-containing materials influence UHPC’s mechanical properties. Notably, fibers with distinct micro-morphologies exhibit complex nonlinear relationships between their content variations and the resulting mechanical performance of UHPC [28,29].

The mechanical properties of ultra-high-performance concrete (UHPC) demonstrate complex correlations with its constituent materials due to the combined effects of both macro- and micro-scale factors [30]. This complexity underscores the importance of developing predictive models based on UHPC’s macro- and microstructural characteristics. As illustrated in Table 1, conventional empirical formulas can effectively describe the compressive strength behavior of specific UHPC mixtures, offering excellent interpretability and high goodness of fit. However, their applicability remains limited to particular UHPC formulations.

Table 1 presents studies on the prediction of UHPC compressive strength using traditional empirical formulas. These empirical formulas typically consider only a single or a limited number of influencing factors (such as porosity or curing age), demonstrating relatively high goodness-of-fit values (*R*^2^ ≥ 0.87) under specific material conditions. However, such studies often fail to account for the effects of multiple factors, resulting in significantly reduced prediction accuracy when such models are applied to more complex practical engineering materials.

To develop a comprehensive predictive model for the compressive strength of various UHPC formulations, it is essential to establish a modeling framework that incorporates multiple influencing factors and demonstrates capability in processing both experimental and publicly available datasets. Compared with conventional statistical techniques, machine learning and deep learning approaches exhibit superior performance in accurately predicting material properties by leveraging large-scale datasets [35,36]. Research has demonstrated the significant potential of these advanced computational methods in predicting concrete compressive strength, with numerous studies reporting satisfactory predictive performance [37,38].

Notably, while machine learning methods demonstrate satisfactory predictive accuracy and stability for UHPC mechanical properties using macroscopic composition information [39,40,41], most conventional machine learning and classical statistical approaches fail to account for microstructural characteristics. Unlike tabular data at the macroscopic level, microstructural investigation typically involves large volumes of image data representing structural features, which conventional machine learning methods struggle to process effectively for feature extraction.

Compared with traditional machine learning methods, deep learning approaches such as CNNs demonstrate superior capability in processing image data and extracting critical information from large volumes of images, showing significant application value in fields like agriculture and transportation [42,43]. However, for UHPC applications where macroscopic information primarily exists in tabular form rather than images, CNN-based deep learning methods exhibit notably inferior performance in handling tabular data when compared to machine learning techniques like XGBoost [44].

Consequently, developing CNN-based deep learning models for UHPC compressive strength prediction that maintain high accuracy when processing tabular data representing macroscopic properties would be of significant importance. Such advancement would facilitate the construction of more accurate predictive models by integrating both macro- and micro-scale experimental data in future research.

This paper proposes a CNN-based approach for predicting UHPC compressive strength. The method comprehensively accounts for the material and preparation condition variability arising from different application scenarios while effectively utilizing both existing research data and practical engineering experience. By employing material composition parameters and curing conditions as input features for CNN training and analysis, the proposed approach not only offers operational simplicity but also effectively captures key characteristics influencing UHPC compressive strength, including silica fume content and fiber volume fraction.

The main contributions of this paper include the following: (i) The development of a CNN-based predictive model for UHPC compressive strength, which demonstrates excellent accuracy and stability through evaluation metrics including the coefficient of determination (*R*^2^), mean absolute percentage error (MAPE), root mean square error (RMSE), and mean absolute error (MAE). The model exhibits comparable performance to machine learning methods like XGBoost when processing tabular data. (ii) The effective utilization of existing mix proportion data, primarily sourced from UHPC mechanical property studies. By directly incorporating UHPC mix proportions and curing conditions as model inputs, the approach eliminates conversion requirements, thereby enhancing operational practicality without generating additional workload during real-world applications. (iii) The achievement of *R*^2^ > 0.95 on the test set, confirming the model’s capability to reliably predict compressive strength across varying mix proportions and curing durations. With a MAPE < 10% on test data, the CNN model maintains prediction errors within an acceptable range, demonstrating its generalizability. This work provides both methodological and theoretical advancements for UHPC compressive strength prediction, offering valuable insights for future research on UHPC mechanical performance forecasting.

The paper is organized as follows: Section 2 describes the data sources, selected parameters, and the architecture of the constructed CNN model. Section 3 presents the model’s performance on our dataset, including comparative analyses with BP neural networks and XGBoost, along with interpretability validation. Section 4 discusses limitations in the model’s construction and training process, as well as distinctions from similar studies. Finally, Section 5 summarizes the research significance and future perspectives.

## 2. Data Sources and Prediction Model Development

To enhance the operability and predictive accuracy of the CNN-based UHPC compressive strength prediction model in practical applications, this section introduces the model development process and relevant details, as well as the data sources used for model training and validation.

### 2.1. Data Sources and Information Processing

#### 2.1.1. Data Sources

To verify the generalizability and practical application value of the proposed CNN-based UHPC compressive strength prediction model, the model was trained and tested using 219 data samples collected from 19 relevant studies. These studies examined the influence of various factors on UHPC compressive strength, including material composition, fiber morphology, and curing duration. Thus, the data obtained from these studies encompasses a wide range of factors, ensuring the generalizability of the prediction model. Table 2 lists the basic information of these studies. It should be noted that each study varied significantly in its focus on UHPC compressive strength factors. Even when the research themes were similar, considerable differences existed in the specific material categories, such as whether fibers were added and the shapes of those fibers. Therefore, Table 2 only highlights the main factors affecting UHPC compressive strength in each study.

#### 2.1.2. Data Processing

Given the wide variety of materials used in existing studies, constructing a prediction model with specific materials as input indicators could reduce generalizability and increase complexity in practical applications. To address this issue, the CNN prediction model in this paper uses interpretable indicators based on existing concrete-mix-ratio knowledge, making it suitable for predicting the compressive strength of concrete prepared with different materials [62]. Therefore, this paper selects cement content, water content, silica fume content, silicon powder content, silica sand content, and high-range water reducer content as the primary components for each UHPC experiment, with other material components calculated relative to cement content. Additionally, since fiber is often added to improve UHPC compressive strength, fiber volume fractions of silica, carbon, and other types are included as basic input variables. It is important to note that although the fiber shape can influence the compressive strength performance of UHPC [29], most existing studies did not consider the effect of fiber morphology, making it difficult to gather sufficient data to support the model training. Thus, the effect of fiber shape is not considered in this paper. Additionally, existing research has shown that the curing duration significantly affects the compressive strength of UHPC [34], and therefore, curing duration is also included as a basic input variable in this paper. Furthermore, since different studies focus on different aspects of UHPC’s mechanical properties, the specimen shape used for testing (cube or cylinder) will also impact the measured compressive strength. As such, the specimen shape is also treated as a basic input variable.

It should be noted that although fiber dimensions and aggregate characteristics (e.g., particle size) may influence UHPC performance, the input parameters selected in this paper were based on the following considerations: (1) sufficient data could be obtained from the existing literature to facilitate model training and (2) preliminary verification demonstrated that the current parameters could effectively predict compressive strength (see Section 3 for results). Future studies investigating the influence of UHPC microstructural properties will incorporate more detailed parameters to further optimize the model.

**Figure 1:**
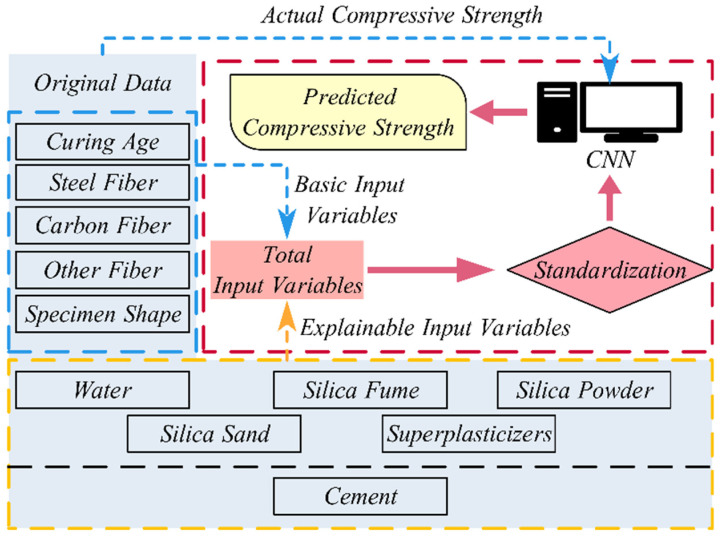
Data preprocessing and usage flow.

In summary, this paper selects cement content; water content; silica fume content; silicon powder content; silica sand content; high-range water reducer content; the fiber volume fraction for silica fibers, carbon fibers, and other types of fibers; curing duration; and the specimen shape as the basic input variables. The cement content, water content, silica fume content, silica powder content, silica sand content, and superplasticizer content are expressed as ratios relative to cement content, resulting in five interpretable input indicators for model training. The remaining basic input variables are also included, yielding a dataset with 10 basic input variables and corresponding compressive strength values. The entire data collection and processing procedure is outlined in Figure 1.

In Figure 1, it is important to note that the basic input variables have different dimensional scales, so the magnitude of their data can vary. If the data were input directly without preprocessing, it could lead to poor model training results. Therefore, the data corresponding to these 10 basic input variables must undergo normalization preprocessing. The standardization formula for a given input variable’s sample data is given below:

(1) $$Z_{i}=\frac{x_{i}-\mu }{\sigma }$$

Here, $Z_{i}$ is the standardized value, $x_{i}$ is the original data value, μ is the mean of the input variable, and σ is the standard deviation of the input variable.

Taking a representative sample as an example, the raw data comprises 10 fundamental input parameters: curing age (CA), steel fiber volume (SFV), carbon fiber volume (CFV), other-fiber volume (OFV), specimen shape (Shape), water–cement ratio (W/C), silica sand–cement ratio (SS/C), silica powder–cement ratio (SP/C), silica fume–cement ratio (SF/C), and superplasticizer–cement ratio (S/C). The original unstandardized data are presented in the first column of Table 3. The mean values and standard deviations for each parameter, calculated from the complete dataset, are shown in columns 2 and 3, respectively. For each data entry, the processed value (dealt data) can be obtained using Equation (1). It should be noted that in the currently collected sample data, no studies had employed fibers other than steel and carbon fibers, resulting in consistently zero values for the OFV parameter after processing. Therefore, Equation (1) is not applicable for this particular parameter.

### 2.2. Prediction Model Development and Detailed Information

#### 2.2.1. CNN Model Structure

**Figure 2:**
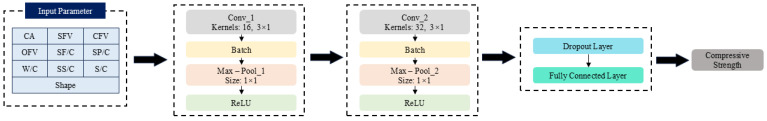
CNN model structure.

Given the significant variation in the materials between samples in this paper, a convolutional structure was added to the model. Each convolutional structure includes a convolutional layer (with a kernel size of 3 × 1 × 1), a pooling layer (with a kernel size of 1 × 1), a batch normalization layer, and a ReLU activation function layer. The collected data enters the model through the input layer, and after basic training through two convolutional structures, redundant data are discarded using a dropout layer. The remaining data are merged in the fully connected layer, and finally, a regression layer is used for model training and data prediction. The structure of the CNN model is shown in Figure 2.

For each convolutional structure, the input data undergoes standardization prior to processing. The data first passes through convolutional layers for feature extraction, employing 16 kernels (3 × 1 size) in the first layer and 32 kernels (3 × 1 size) in the second layer. Batch normalization is then applied to the extracted features to mitigate excessive data variance after convolution that could adversely affect subsequent computations. Pooling layers subsequently reduce data dimensionality, decreasing the computational load, while ReLU activation functions enhance the model’s capability to capture nonlinear characteristics in new data. This processing sequence ensures optimal feature extraction while minimizing redundant computations, thereby improving training efficiency.

#### 2.2.2. Detailed Information on CNN Model

Under the framework of the model, in addition to the input parameters, there are hyperparameters that need to be configured, primarily including the loss function of the model, the learning rate for controlling gradient variations, the maximum number of training epochs, and other relevant parameters.

The loss function quantifies the discrepancy between the predicted values, obtained by processing the input parameters of each sample group through the model, and the corresponding true values of UHPC. The formulation of the loss function significantly influences the training outcomes of the CNN model. To maximize the model’s training accuracy while preventing excessively large errors in individual predictions, this paper adopts the mean squared error (MSE) as the loss function, which is defined as follows:

(2) $$L^{N}=\frac{1}{N}\sum_{i=1}^{N}{\left(y_{i}-{\hat{y}}_{i}\right)}^{2}$$

Here, N is the total number of samples, $y_{i}$ is the true value of the *i*-th sample, and ${\hat{y}}_{i}$ is the predicted value of the *i*-th sample.

In the TensorFlow framework of Python 3.12, convolutional neural network optimizers employ various criteria for optimizing model parameters. This paper adopts the stochastic gradient descent (SGD) method as the optimizer.

**Figure 3:**
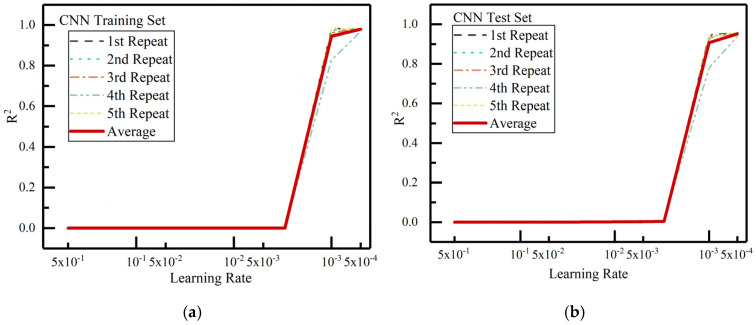
Influence of learning rate on CNN model fit. (**a**) Influence of learning rate on CNN model fit (training set). (**b**) Influence of learning rate on CNN model fit (test set).

To accelerate convergence and avoid large updates leading to instability, the learning rate was adjusted starting from 0.5, gradually decreasing until the optimal value was found. The effect of the learning rate on the model’s fit during training and testing is illustrated in Figure 3.

Figure 3 shows the effect of the learning rate on the CNN model’s prediction performance. As observed, when the learning rate is larger than 0.001, the model struggles to converge, resulting in unstable training. However, at a learning rate of 0.001, although the model performs well in terms of prediction accuracy on average, some individual experiments show lower goodness-of-fit values (below 0.8), indicating instability in the model’s predictions. When the learning rate is decreased to 0.0005, the model demonstrates both higher prediction accuracy and stability, with goodness-of-fit values exceeding 0.95 in five repeated experiments for both the training and testing sets. Thus, the learning rate of 0.0005 was selected for training, with a batch size of 30 samples, meaning a maximum of 5000 training iterations.

With reference to existing studies employing deep learning methods for predicting concrete compressive strength [62], satisfactory model performance has been achieved when the dataset is partitioned with 80% for training and 20% for testing. Therefore, this paper adopts the same data splitting criterion. To validate and evaluate the predictive performance of CNN for UHPC compressive strength while ensuring experimental reproducibility, the CNN model was constructed, trained, and tested using the TensorFlow framework based on Python 3.12.

#### 2.2.3. Evaluation Metrics for CNN Model Training

To comprehensively evaluate the model’s predictive performance, this paper employs the coefficient of determination (*R*^2^) as the primary metric for assessing prediction accuracy across all samples. The *R*^2^ value, calculated using Equation (3), quantifies the model’s ability to accurately represent the majority of the dataset.

(3) $$R^{2}=1-\frac{\sum_{i=1}^{N}{\left(Predicted_{i}-Actual_{i}\right)}^{2}}{\sum_{i=1}^{N}{\left(Actual_{i}-\bar{Actual}\right)}^{2}}$$

Here, *Predicted_i_* represents the predicted compressive strength for the *i*-th sample in the model, *Actual_i_* represents the actual compressive strength of the *i*-th sample, and $\bar{Actual}$ represents the mean of all the actual compressive strength values for the samples.

In addition to the coefficient of determination, this paper employs three additional evaluation metrics—the root mean square error (RMSE), mean absolute percentage error (MAPE), and mean absolute error (MAE)—to assess the predictive performance of the CNN model for UHPC compressive strength. These metrics collectively evaluate the stability of the model’s predictions, with their respective computational formulas provided in Equations (4)–(6).

(4) $$MAPE=\frac{1}{N}\sum_{i=1}^{N}\left|\frac{Actual_{i}-Predicted_{i}}{Actual_{i}}\right|\times 100\%$$

(5) $$RMSE=\sqrt{\frac{1}{N}\sum_{i=1}^{N}{\left(Actual_{i}-Predicted_{i}\right)}^{2}}$$

(6) $$MAE=\frac{1}{N}\sum_{i=1}^{N}\left|Actual_{i}-Predicted_{i}\right|$$

## 3. Model Prediction Results and Analysis

### 3.1. CNN Model Prediction Performance

For each training iteration in this paper, an identical dataset partitioning method was employed. As evidenced by the results in Figure 3, when the learning rate was set to 0.0005, multiple experimental trials consistently achieved goodness-of-fit values exceeding 0.9. Consequently, further hyperparameter tuning was deemed unnecessary. The evaluation metric values from one representative model training experiment are presented in Table 4.

As shown in Table 4, the CNN model demonstrated high accuracy and stability. On the training set, the *R*^2^ value was 0.982, and the MAPE was only 2.423%, indicating excellent prediction performance. On the test set, the model achieved an *R*^2^ of 0.959, with a MAPE of 5.550%, demonstrating strong generalizability and good stability for unseen data.

**Figure 4:**
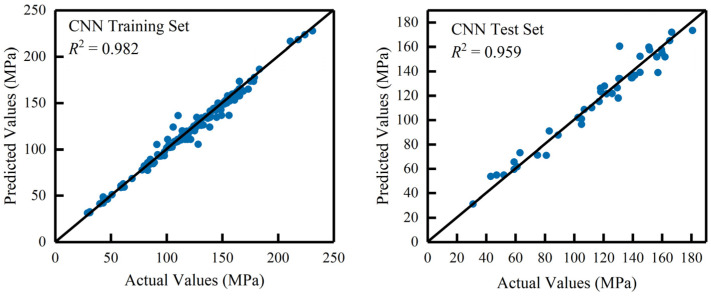
Relationship between predicted and actual compressive strength (training and test sets).

To visually assess the model’s performance, Figure 4 compares the predicted and actual values of UHPC compressive strength for both the training and test sets. The vertical axis represents the predicted compressive strength while the horizontal axis shows the actual compressive strength values. As seen in the Figure, the data points are clustered close to the diagonal line, indicating that the model’s predictions align well with the actual values. This further confirms that the CNN model can accurately predict UHPC compressive strength.

**Figure 5:**
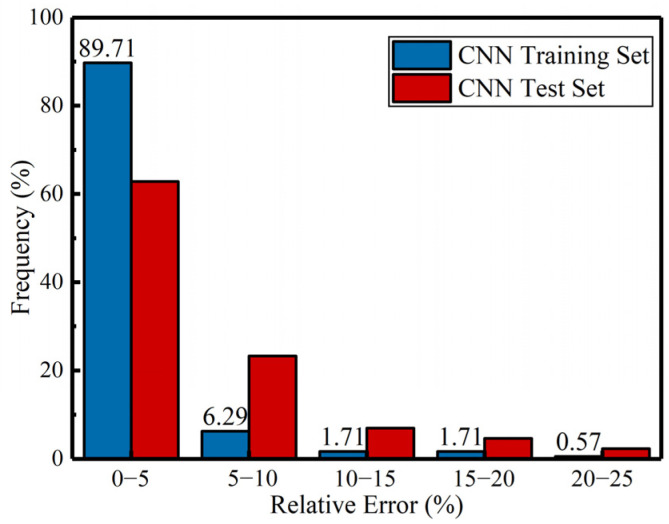
Relative error distribution for CNN predictions (training and test sets).

In addition to the *R*^2^ values, relative error histograms were plotted to visually assess the distribution of prediction errors in both the training and test sets. Figure 5 shows the relative error distribution for both sets. From the analysis, it is evident that the minimum relative error in the training set was 0.03%, and in the test set, it was 0.09%. Moreover, nearly 90% of the samples in the training set had a relative error of less than 5%, and more than 60% of the samples in the test set had a relative error of less than 5%. When a 10% relative error threshold was used, over 95% of the samples in the training set had a relative error of less than 10% while more than 85% of the test set samples fell below this threshold. This further demonstrates the accuracy and stability of the CNN model for predicting UHPC compressive strength. The proportion of samples with relative errors greater than 20% was less than 1% for both training and test sets, highlighting the model’s reliability.

### 3.2. Comparison of CNN with Other Common Machine Learning Methods

To demonstrate the superiority of the proposed CNN prediction model, this paper compares its performance with other prevalent machine learning approaches, including backpropagation neural networks (BPs), multilayer perceptrons (MLPs), and XGBoost, which have demonstrated satisfactory accuracy in processing non-image data. To ensure a fair comparison of different models’ predictive capabilities for UHPC mechanical properties, identical training and testing datasets were employed across all model evaluations. It should be noted that the hyperparameters varied among different models as manual parameter tuning was conducted to optimize each model’s performance on the same dataset.

**Figure 6:**
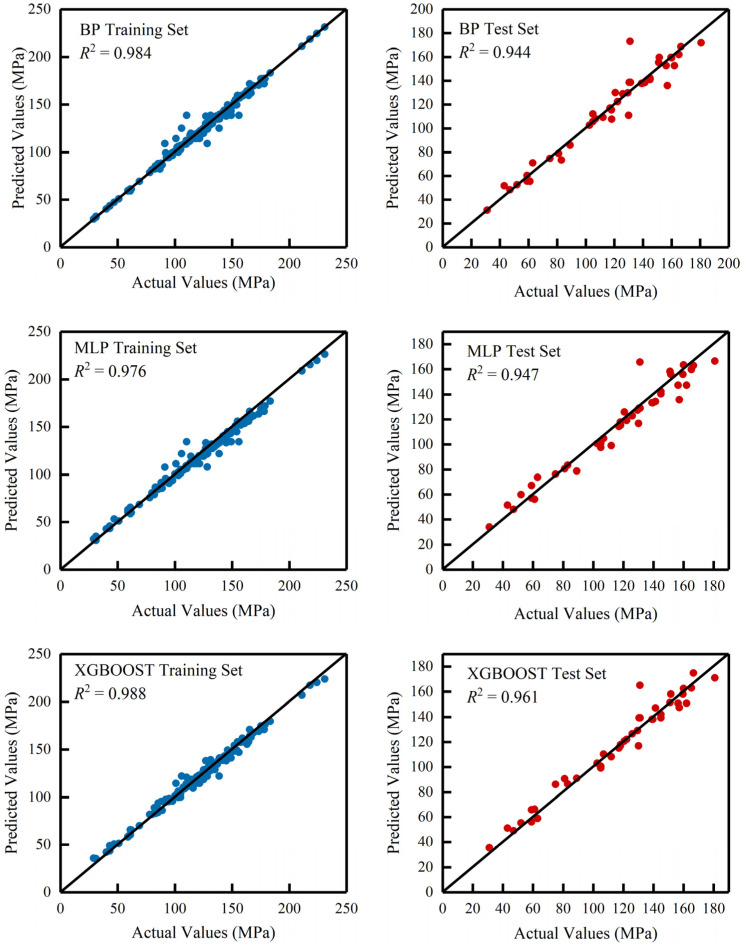
Predictive performance of comparative methods on the identical dataset.

Figure 6 presents the predictive performance of BP neural networks, MLPs, and XGBoost for UHPC compressive strength using data from various sources. The data points for both training and test sets were predominantly distributed near the diagonal. While some test set points from BP and MLP showed moderate deviations from the diagonal, no significant outliers were observed. These results indicate that all three methods achieve acceptable prediction accuracy for UHPC with different material compositions. Notably, XGBoost, being particularly effective for tabular data processing, demonstrates superior performance to CNN when handling exclusively tabular-formatted input data.

**Figure 7:**
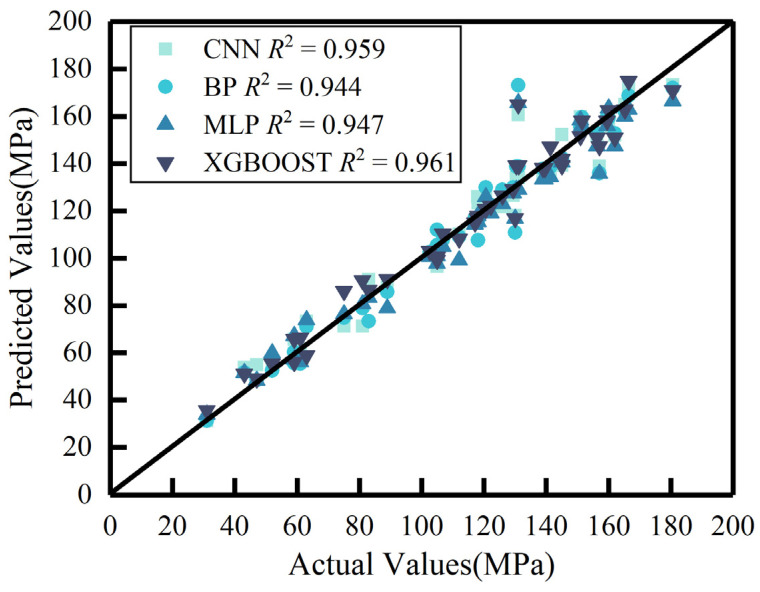
Comparison of predicted versus actual values for different methods in the testing set.

For a direct comparison of the predictive performance between CNN and alternative methods (BP, MLP, XGBoost) in estimating UHPC compressive strength, Figure 7 presents the relationship between predicted and actual values for all four methods within the testing set.

Figure 7 demonstrates the predictive performance of four methods for UHPC compressive strength through their data point distributions. Most data points clustered around the diagonal for all methods, indicating that both machine-learning- and deep-learning-based predictive models maintain high accuracy when processing tabular data. Accuracy analysis reveals that the CNN shows no significant disadvantage compared to conventional superior methods for tabular data analysis and even maintains comparable performance when handling macro-level UHPC tabular data.

Although visual analysis indicates that both CNNs and other models demonstrate satisfactory predictive performance, additional metrics beyond overall goodness of fit should be examined to evaluate whether any model produces significant errors at specific data points.

**Figure 8:**
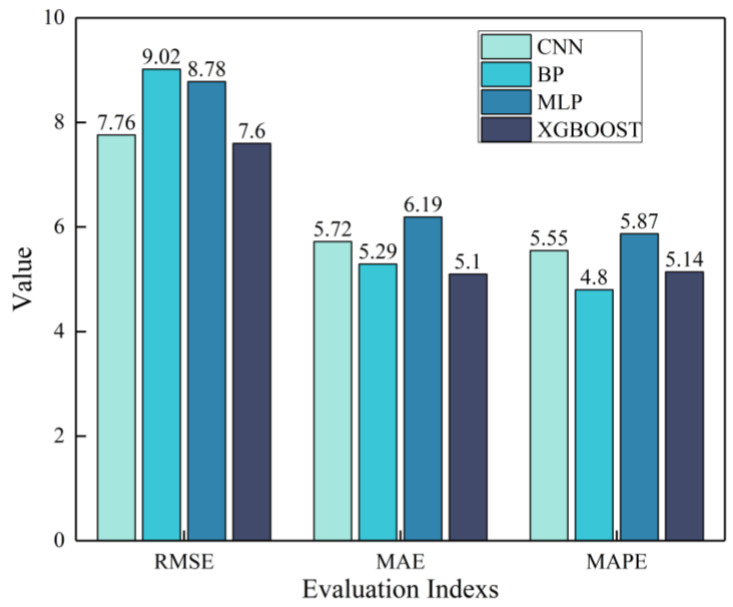
Comprehensive performance comparison between CNN and comparative methods.

For a comprehensive comparison of predictive performance between CNNs and other models, this paper compares four models using the RMSE, MAE, and MAPE metrics. As shown in Figure 8, the CNN exhibited lower RMSE values in the testing set compared to BP and MLP while the BP outperformed the CNN in terms of the MAE and MAPE. Multi-metric analysis revealed that the CNN slightly surpassed the MLP across all evaluated indicators, yet showed inferior performance to XGBoost. The comparison with the BP yielded mixed results, with each method demonstrating relative advantages in different metrics.

For CNN and BP models that demonstrate respective advantages across multiple metrics, the significantly lower RMSE of the CNN’s test set compared to the BP indicates that the CNN produces smaller maximum deviations between predicted and actual values for individual samples. This suggests that the CNN exhibits better stability in practical applications, with minimal bias across various UHPC types. In contrast, the BP’s higher RMSE implies potentially larger errors when processing certain input data.

Regarding the MAE and MAPE metrics where the BP slightly outperformed the CNN, two observations emerge. First, both models achieved MAPE values below 10% on the test set, demonstrating satisfactory accuracy across diverse UHPC types. Second, although the BP may have generated larger errors for outliers, it maintained acceptable precision for most data points. Therefore, when dealing with tabular data reflecting UHPC’s macroscopic characteristics, the BP remains reasonably effective.

Regarding the MLP and XGBoost, XGBoost is widely recognized as superior to the CNN for processing tabular data, which is consistent with our findings showing XGBoost’s better performance across all evaluation metrics. Both methods require hyperparameter tuning based on training datasets. Although this paper did not investigate optimal hyperparameter adjustment mechanisms (potentially limiting MLP’s full performance), the MLP nevertheless demonstrated satisfactory prediction accuracy and stability.

In summary, both the CNN model and other conventional models demonstrate effective predictive capabilities for the compressive strength of various UHPC types. Notably, the CNN maintains consistent performance with minimal large prediction errors, exhibiting good stability even when processing only tabular data. Although existing studies and our experimental results have confirmed that machine learning methods like XGBoost show superior performance with tabular data, the CNN nevertheless demonstrates comparable effectiveness without significant disadvantages.

Considering practical applications requiring the image analysis of UHPC’s microstructural characteristics, conventional machine learning methods like XGBoost and the BP would face challenges in specialized processing. Therefore, CNN models are expected to demonstrate superior performance when extended to prediction scenarios incorporating more material characteristics of UHPC compressive strength.

### 3.3. Interpretability of the CNN Model

**Figure 9:**
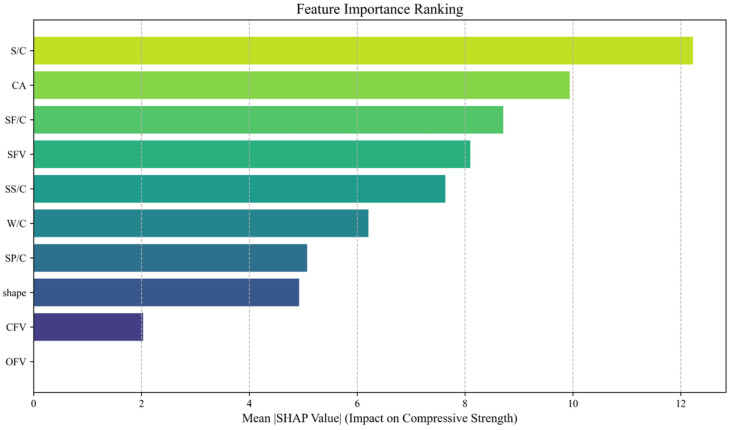
Contribution ranking of input parameters in the model.

To elucidate the operational mechanism of the developed CNN model, the SHapley Additive exPlanations (SHAP) method was employed to quantify the contribution of input parameters (e.g., W/C ratio) to UHPC compressive strength. Specifically, the SHAP library in Python was utilized to analyze the CNN model constructed using the TensorFlow framework. The resulting contribution rankings of input parameters in the CNN model are presented in Figure 9.

In Figure 9, all parameters are ranked in descending order of their influence magnitude. Eight parameters exhibit influence scores exceeding 3, indicating significant impacts on the model’s predictions. In contrast, the CFV and OFV demonstrate minimal influence, suggesting negligible effects on the outcomes. This observation stems from the limited representation of carbon fiber and other fiber data in the collected samples, resulting in insufficient training impact. The remaining eight parameters show substantial influence, confirming the appropriateness of the selected parameters in this paper.

**Figure 10:**
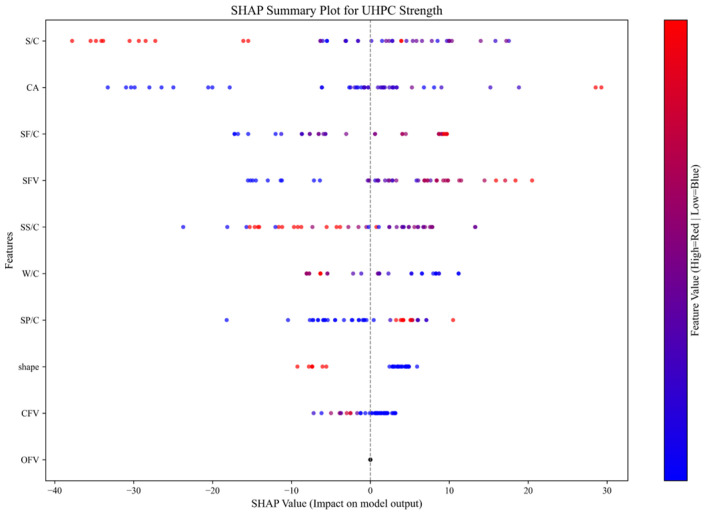
SHAP summary plot.

To quantify the influence of input parameters on the CNN model’s prediction of UHPC compressive strength, SHAP summary plots were employed to visualize the contribution of each feature, thereby facilitating the further analysis of their impact mechanisms, as illustrated in Figure 10.

Figure 10 presents the parameters ranked in descending order of contribution, consistent with the ordering in Figure 9. Red data points indicate higher parameter values while blue represents lower values. When red points predominantly cluster in the SHAP value > 0 region (or blue points in SHAP value < 0), this demonstrates strong positive correlation with UHPC compressive strength predictions. Conversely, the opposite distribution suggests negative correlation.

Figure 10 demonstrates positive correlations between the CA, SF/C, SFV, SP/C, and UHPC compressive strength, indicating that within the model’s training data range, longer curing ages and higher contents of silica fume, silica powder, and steel fiber correspond to increased compressive strength. This observed trend aligns with findings from existing studies.

Conversely, W/C and shape exhibit negative correlations with UHPC compressive strength. Specifically, higher water–cement ratios correspond to lower compressive strengths within the model’s training data range. Regarding the specimen shape, smaller shape values (where 0 represents cubic specimens and 1 denotes cylindrical specimens in this paper) are associated with higher compressive strengths. This finding is consistent with empirical observations in engineering practice, where cubic specimens typically yield greater compressive strength values than cylindrical ones for the same concrete mixture.

Notably, some parameters do not exhibit typical positive or negative correlations. Taking S/C as an example, purple data points (indicating intermediate parameter values) cluster in the SHAP value > 0 region while blue points (low values) are concentrated in the SHAP value < 0 area, and red points (high values) appear further left than blue ones. This pattern suggests that within the model’s training data range, (1) lower superplasticizer content corresponds to reduced UHPC compressive strength, (2) strength increases with a rising content, but (3) exceeding a certain threshold, strength declines rapidly.

This phenomenon may stem from multiple factors: first, excessive superplasticizer addition in practice genuinely compromises concrete strength; second, the dataset may lack sufficient samples with overdosed superplasticizers, leading to suboptimal model training. Nevertheless, the overall trend remains consistent with established engineering experience.

In summary, the SHAP analysis demonstrates that within the dataset collected for this paper, eight out of ten input parameters (80%) in the CNN model significantly influence UHPC compressive strength predictions. Moreover, the correlation patterns between these parameters and compressive strength align well with established relationships observed in practical engineering applications regarding material properties, construction techniques, and UHPC strength performance. These findings validate the rationality of the selected parameter system and confirm the interpretability of the CNN model, thereby providing valuable references for engineering applications of CNNs in UHPC compressive strength prediction.

### 3.4. Stability of the CNN Model

**Figure 11:**
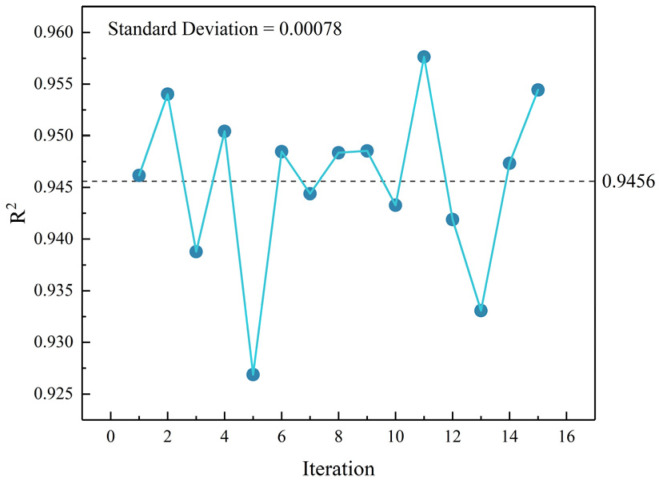
Performance evaluation of the CNN model on identical datasets.

To verify the stable performance of the developed CNN model with fixed hyperparameters across both training and testing datasets, we conducted 15 independent runs using the identical training set with hyperparameters specified in Section 2. Figure 11 presents the results of these repeated experiments, demonstrating the model’s consistent performance in each execution.

Figure 11 demonstrates that the CNN model with fixed hyperparameters consistently achieves similar goodness-of-fit values across all runs. Specifically, the 15 experimental trials yielded an average *R*^2^ of 0.9456 with a standard deviation of 0.00078, indicating stable model performance under the current hyperparameter configuration for the given dataset.

It should be noted that this paper first partitioned the complete dataset and then manually optimized the hyperparameters through the comparative analysis of running results, ultimately achieving satisfactory model performance. Should the dataset be modified, corresponding hyperparameter adjustments would be required to maintain optimal model effectiveness.

When employing random partitioning to generate multiple training–test set combinations, the CNN model struggles to achieve optimal performance across all data splits, even with independent hyperparameter optimization for each split. This limitation primarily stems from two factors: (1) the limited sample size of the complete dataset (n = 219), which prevents the randomly partitioned subsets from adequately representing all features, and (2) significant heterogeneity among data sources, resulting in substantial feature variations across samples. Consequently, randomly partitioned training sets may exhibit considerable divergence, meaning a model performing well on one training set may not be generalized effectively to others.

**Figure 12:**
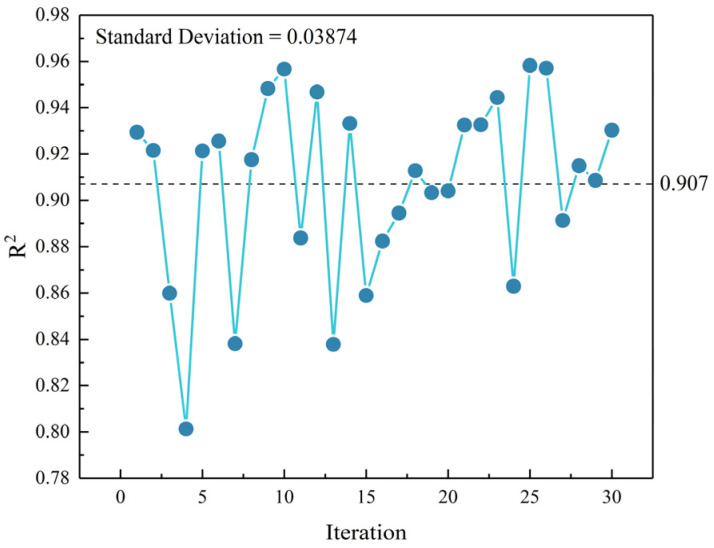
Performance evaluation of the CNN model across multiple dataset configurations.

To better illustrate this phenomenon, we generated 30 distinct training–test set pairs using random partitioning, differing from those in Figure 11. The CNN model with the architecture and hyperparameters specified in Section 2 was then evaluated on these 30 dataset combinations, with the results presented in Figure 12.

As shown in Figure 12, the CNN model exhibits significantly degraded predictive performance when applied to different data partitions, with the average *R*^2^ decreasing to 0.907 and the standard deviation increasing to 0.03874 across multiple runs. These results indicate that the limited sample size (n = 219) and compositional diversity of UHPC specimens prevent individual training sets from adequately covering the material’s feature space, leading to substantial variations between datasets. Consequently, the observed performance fluctuations across different training–test set combinations primarily reflect insufficient sample data volume rather than inherent limitations of the CNN architecture.

In conclusion, the stability analysis presented in Figure 11 demonstrates that the CNN model achieves reliable and consistent predictive accuracy with the current dataset of over 200 samples. To extend the model’s applicability to a broader variety of UHPC formulations, it is essential to expand the dataset by incorporating additional specimens with diverse mix proportions and constituent materials, which would significantly enhance the model’s generalization capability.

## 4. Discussion

The CNN model developed in this paper accurately predicted UHPC compressive strength using 10 input parameters from tabular data comprising 219 samples, achieving a maximum test-set *R*^2^ of 0.959. The model demonstrated excellent stability, with consistent performance across 15 consecutive runs (*R*^2^ = 0.9456 ± 0.00078).

It is noteworthy that prior to this investigation, existing studies had employed both machine learning and deep learning approaches to analyze concrete compressive strength and related properties. Table 5 presents a comparative analysis between selected previous studies and the current research findings.

Table 5 compares the performance of CNNs and XGBoost across different studies, revealing the influence of methodological characteristics and data scale on prediction accuracy:

(1) Methodological characteristics: For tabular data-based predictions, the performance difference between CNNs (*R*^2^ = 0.959) and XGBoost (*R*^2^ = 0.961) was merely 0.2%, indicating that despite the CNN being originally designed for image processing, it remains competitive in handling material-science tabular data.

(2) Data scale: While the current dataset (size = 219) yields stable results, expanding the sample size (e.g., to 350+ as in studies [41,62]) could further enhance the model’s predictive stability for diverse UHPC mix designs.

It should be further noted that the CNN can directly process UHPC microstructure images without manual intervention, giving it a unique advantage over XGBoost in future studies incorporating microstructural analysis—compensating for its marginal disadvantage in tabular data processing.

However, it is noteworthy that no study has yet developed a CNN-based microstructural feature recognition model with broad applicability to diverse UHPC compositions. Consequently, the future application of CNN models for UHPC compressive strength prediction presents both opportunities and challenges:

(1) Data fusion capability for macro–micro characteristics: CNNs can potentially integrate microscopic feature images with conventional tabular data through their image processing capacity, establishing correlations between macro- and microstructural features to enhance prediction accuracy across varying UHPC formulations;

(2) Data format inconsistency complicating CNN training: Discrepancies in recording standards across studies pose significant challenges. For macroscopic features, some studies have documented detailed parameters (e.g., fiber morphology) while others only record basic composition data. Microscopic features exhibit further variability in image resolution and clarity due to equipment differences, substantially increasing feature extraction difficulty for CNNs.

## 5. Conclusions

This paper has proposed a convolutional-neural-network-based model for predicting UHPC compressive strength using ten fundamental input parameters (including mix proportions and curing information). The model was trained and tested on 219 datasets obtained from existing studies, yielding the following key findings:

(I) The proposed CNN model demonstrates high predictive accuracy, achieving *R*^2^ = 0.959 and MAPE = 5.55% on the test set. SHAP analysis has confirmed the model’s interpretability while performance comparison has revealed its competitiveness with XGBoost (*R*^2^ = 0.961), the optimal performer for tabular data processing. Furthermore, the model exhibits excellent operational stability, as evidenced by the low standard deviation (0.00078) across 15 consecutive runs.

(II) The CNN model developed in this paper shows promising potential for practical applications. Implemented using Python’s TensorFlow framework, the model offers engineer-friendly coding accessibility. However, its practical implementation requires further investigation due to the current limitation of UHPC test data availability.

(III) The CNN model in this paper exhibits limitations in both dataset coverage and sample size (n = 219). Significant prediction accuracy fluctuations were observed when using different training sets (average *R*^2^ decreased to 0.907 across 30 random partitions), indicating that the current data volume is insufficient to ensure model stability. Particularly noteworthy is the dataset’s lack of UHPC formulations containing novel fiber materials (e.g., carbon fibers) and certain admixtures, which limits the model’s predictive capability for these advanced materials. Future studies should expand the dataset to incorporate these emerging fiber materials.

The predictive model developed in this paper can utilize existing UHPC formulation experience to estimate compressive strength for different mix designs, showing potential for future integration of both macro- and microstructural features to achieve more accurate predictions. However, it should be noted that the current paper did not establish an effective strategy for adjusting hyperparameters (e.g., learning rate) during model development and training. The simplified search method employed may prove inadequate for hyperparameter optimization in complex scenarios. Particularly when applied to modified datasets, manual hyperparameter tuning would introduce uncertainty and increase computational costs. Therefore, subsequent research should focus on developing appropriate hyperparameter optimization algorithms to enhance the model’s adaptability across diverse datasets. Such improvements would enable faster convergence and consequently improve both the prediction accuracy and applicability range of the model.

## Figures and Tables

**Table 1:** UHPC compressive strength prediction models and goodness of fit.

Features	Model	*R* ^2^
Porosity [31]	σ=144.77−5.90P	0.91
Recycled Concrete Powder/Recycled Paste Powder Content [32]	$f_{c,RCP}=f_{c0}-0.352P_{RCP}$	-
Steel Fiber [33]	$\sigma_{c}=k_{1}\sigma_{m}\left(1-V_{f}\right)+k_{2}\tau l_{f}/d_{f}\times \eta_{\theta }V_{f}$	0.87
Age [34]	$lg\left(f_{c}\right)=0.21lg\left(x\right)+1.71$	0.98

**Table 2:** Data sources and main factors in research.

Author	Main Variable
Shuo Zhao et al. [45]	Plastic synthetic fiber, steel fiber
Antonija Ocelić et al. [46]	Recycled tire steel fiber, carbon fiber
Weiwen Li et al. [47]	Fiber-reinforced polymer
J.J. Wang et al. [48]	Fiber-reinforced polymer
S.S. Zhang et al. [49]	Steel fiber volume fraction
Dingqiang Fan et al. [50]	Steel fiber volume fraction
Junqing Xue et al. [51]	Steel fiber volume fraction
Shack Yee Hiew et al. [52]	Steel fiber volume fraction
Xin TIAN et al. [28]	Steel fiber volume fraction, fiber shape
Zilong Tang et al. [53]	Steel fiber volume fraction
Samaneh Khaksefidi et al. [54]	Concrete strength
Jia-Xiang Lin et al. [55]	Polypropylene fiber, polyoxymethylene fiber
Yu Rui et al. [56]	Steel fiber, polyoxymethylene fiber
Shunan Wang et al. [57]	Steel fiber volume fraction, fiber aspect ratio
Shunan Wang et al. [58]	Steel–polypropylene hybrid fiber
Mostafa Hassan et al. [59]	Steel fiber volume fraction
Kewei Liu et al. [29]	Fly ash, blast furnace slag, steel fiber type
Yang Zhang et al. [60]	Fiber volume fraction
Zefang Wang et al. [61]	Steel fiber volume fraction, shear connector

**Table 3:** Example of data standardization processing.

Parameter	Original Data	Mean Value	Standard Deviation	Dealt Data
CA	28	23.018	22.705	0.219
W/C	0.230	0.284	0.103	−0.518
SS/C	1.589	1.384	0.676	0.303
SP/C	0	0.323	0.503	−0.642
SF/C	0.212	0.255	0.160	−0.267
S/C	0.027	0.048	0.020	−1.008
SFV	0.500	1.059	0.979	−0.572
CFV	1.500	0.348	0.676	1.703
OFV	0	0.000	0.000	0.000
shape	0	0.361	0.480	−0.751

**Table 4:** Summary of the performance metrics of the CNN model on both the training and test datasets.

Data Set	Performance Measures			
	*R* ^2^	RMSE (MPa)	MAPE (%)	MAE (MPa)
Training set	0.982	4.809	2.423	2.765
Test set	0.959	7.762	5.550	5.724

**Table 5:** Comparative analysis between current and previous research findings.

Method	*R*^2^ (This Paper)	Multimodal Extensibility	*R*^2^ (From Different Studies)
CNN	0.959	Native image input support, no feature engineering needed	0.967 (conventional concrete) [62]
XGBOOST	0.961	Requires manual image feature extraction	0.973 (UHPC) [41]

## Data Availability

The original contributions presented in this study have been included in the article. Further inquiries can be directed to the corresponding author.
